# Ultrasound, PET/CT or temporal artery biopsy for giant cell arteritis? A prospective diagnostic accuracy study (the GAME‐study)

**DOI:** 10.1111/aos.70114

**Published:** 2026-03-07

**Authors:** Michael S. Hansen, Lene Terslev, Uffe M. Døhn, Viktoria Fana, Jane M. Brittain, Anne K. Wiencke, Steffen Heegaard, Mads R. Jensen, Oliver N. Klefter, Yousif Subhi, Steffen Hamann

**Affiliations:** ^1^ Department of Ophthalmology Rigshospitalet Copenhagen Denmark; ^2^ Department of Clinical Medicine University of Copenhagen Copenhagen Denmark; ^3^ Department of Rheumatology and Spine Diseases Rigshospitalet Copenhagen Denmark; ^4^ Department of Clinical Physiology and Nuclear Medicine Rigshospitalet Copenhagen Denmark; ^5^ Department of Pathology Eye Pathology Section, Rigshospitalet Copenhagen Denmark; ^6^ Department of Clinical Physiology and Nuclear Medicine Bispebjerg Hospital Copenhagen Denmark; ^7^ Department of Clinical Research University of Southern Denmark Odense Denmark; ^8^ Rothschild Foundation Hospital Paris France

**Keywords:** 2‐[^18^F]FDG PET/CT, Giant cell arteritis, paracentral acute middle maculopathy, temporal artery biopsy, ultrasound

## Abstract

**Purpose:**

To investigate the diagnostic performance of ultrasound, 2‐deoxy‐2‐[^18^F]fluoro‐D‐glucose positron emission tomography/computed tomography (2‐[^18^F]FDG PET/CT) and temporal artery biopsy (TAB) in giant cell arteritis (GCA).

**Methods:**

This was a prospective single‐centre diagnostic accuracy study (ClinicalTrials.gov NCT05248906). One hundred and ten patients suspected of GCA were considered, of which 103 were analysed, 100 had a final diagnosis and 58 had complete data for all modalities. Ultrasound, 2‐[^18^F]FDG PET/CT and TAB were performed after clinical examination and blood sampling. The main outcome included diagnostic accuracy measures with final clinical expert diagnosis at 6‐month follow‐up as the comparator.

**Results:**

Sixty‐one patients had GCA, 39 had non‐GCA and 3 had inconclusive. The sensitivities were 82% (95% CI: 66%–92%), 74% (95% CI: 57%–87%) and 68% (95% CI: 51%–83%) for ultrasound, 2‐[^18^F]FDG PET/CT and TAB, respectively. The corresponding specificities were 90% (95% CI: 68%–99%), 100% (95% CI: 83%–100%) and 100% (95% CI: 83%–100%), the positive predictive values 94% (95% CI: 81%–98%), 100% (95% CI: 88%–100%) and 100% (95% CI: 87%–100%), the negative predictive values 72% (95% CI: 57%–84%), 67% (95% CI: 54%–77%) and 63% (95% CI: 51%–73%) and accuracies 85% (95% CI: 73%–93%), 83% (95% CI: 71%–91%) and 79% (95% CI: 67%–89%). When ultrasound was inconclusive, 2‐[^18^F]FDG PET/CT had a higher sensitivity of 75% (95% CI: 35%–97%) vs. 38% (95% CI: 9%–76%) for TAB, with equal specificity of 100% (both 95% CI: 69%–100%). Results were inconclusive in 22 of 99 ultrasounds, 3 of 102 2‐[^18^F]FDG PET/CTs and 4 of 90 TABs.

**Conclusion:**

Ultrasound had high diagnostic accuracy for GCA, supporting this as the first imaging modality in suspected GCA. 2‐[^18^F]FDG PET/CT combined a high diagnostic accuracy with fewer inconclusive results, suggesting it may be used for second‐line testing when ultrasound is inconclusive, reserving TAB for third‐line testing. A fast‐track setup may facilitate efficient diagnostics.

## 1 INTRODUCTION

Giant cell arteritis (GCA) is a vasculitis of large‐size arteries. The disease is feared due to the risk of stroke and blindness, making GCA a medical and ophthalmological emergency. Immediate initiation of glucocorticoid treatment as well as a rapid and accurate diagnosis can reduce the risk of adverse outcomes like visual loss (Vodopivec & Rizzo 3rd, 2018). Establishing a diagnosis of GCA can be difficult because no single test can confirm or rule out the condition with 100% accuracy.

Historically, the diagnostic gold standard for GCA has been a temporal artery biopsy (TAB), which secures a histopathological diagnosis. In the past decades, non‐invasive imaging modalities have been introduced (Schmidt, 2018; Schmidt et al., 1997), allowing for a fast‐track setup for GCA (Diamantopoulos et al., 2016; Monti et al., 2020). Ultrasound and 2‐deoxy‐2‐[^18^F]fluoro‐D‐glucose positron emission tomography/computed tomography (2‐[^18^F]FDG PET/CT) as well as other modalities such as magnetic resonance imaging (MRI) with or without black blood sequences have all been successfully used individually as diagnostic tests for GCA (Bley et al., 2007; Brittain et al., 2023; Hansen et al., 2023; Meller et al., 2009). This resulted in European Alliance of Associations for Rheumatology (EULAR) recommending ultrasound as first‐line diagnostic testing for GCA in 2018 (Hellmich et al., 2020). In 2023, ultrasound or alternatively 2‐[^18^F]FDG PET/CT was recommended (Dejaco et al., 2024). While the evidence for using ultrasound or 2‐[^18^F]FDG PET/CT as individual tests for GCA is solid with high sensitivity (SN) and specificity (SP) (Dejaco et al., 2024; Hansen et al., 2023; Lambæk et al., 2023; Sammel et al., 2019; Schmidt, 2018; Schmidt et al., 1997), only a few studies have combined ultrasound, 2‐[^18^F]FDG PET/CT and TAB on the same cohort of patients with suspected GCA in combination with a thorough neuro‐ophthalmological examination. Rottenburger et al. compared 2‐[^18^F]FDG PET/CT with ultrasound and TAB in a small group of GCA patients, but with emphasis on the temporal artery branches. They found that 2‐[^18^F]FDG PET/CT and ultrasound had equivalent diagnostic accuracy, but that 2‐[^18^F]FDG PET/CT had a reduced performance on the parietal branch (Rottenburger et al., 2021). In addition to the cranial arteries, larger arteries such as the aorta and its proximal branches and the vertebral arteries, in isolation or in combination, are prone to inflammation in GCA (Justesen et al., 2021). Therefore, the ability to examine a larger number of arteries for inflammation throughout the body is important (Tomelleri et al., 2023), and it appears logical to test a multimodal diagnostic approach as recommended by EULAR (Dejaco et al., 2024; Hellmich et al., 2020).

The aim of this study was to apply the combination of ultrasound, 2‐[^18^F]FDG PET/CT and TAB in a multimodal approach on a prospective cohort of patients clinically suspected of GCA. The hypothesis was that 2‐[^18^F]FDG PET/CT could be used for second‐line diagnostics, following ultrasound and reserving TAB only to be used for third‐line diagnostics when ultrasound and 2‐[^18^F]FDG PET/CT are inconclusive or negative and the GCA suspicion remains.

## 2 METHODS

### 2.1 Study design and ethics

This was a single‐centre, diagnostic accuracy study with consecutive sampling. Patients were included from the Department of Ophthalmology and the Department of Rheumatology and Spine Diseases at Rigshospitalet, Denmark, between March 2021 and January 2024 (ClinicalTrials.gov NCT05248906). Ethical approval was given by the Regional Research Ethics Committee (H‐20032069). The ‘GAME‐study’ (Giant Cell Arteritis Multimodal Evaluation) adhered to the tenets of the Declaration of Helsinki and hospital guidelines. All participants gave written informed consent.

### 2.2 Patient eligibility and study cohort

Patients 50 years of age or older and with clinical suspicion of GCA with and without visual symptoms were included. The patients should adhere to the 1990 American College of Rheumatology criteria, including headache, temporal artery abnormality (tenderness or reduced/absent pulse), ESR ≥50 mm/h and abnormal TAB, respectively. The 2022 American College of Rheumatology criteria were not imposed due to the start of inclusion in 2021. Typical clinical symptoms and findings, such as headache, scalp tenderness, jaw and tongue claudication, fever, weight loss and elevated inflammatory biomarkers, were included. Patients were excluded if glucocorticoids had been taken for longer than 1 week within the preceding 6 months or if the patient had a known active cancer.

Included patients underwent initial first visit examination and blood tests. Baseline was defined as the first clinical presentation. Blood samples were preferably drawn at the first clinical suspicion of GCA. The baseline examination was followed by neuro‐ophthalmological examination, ultrasound, 2‐[^18^F]FDG PET/CT and TAB.

### 2.3 Neuro‐ophthalmological examination

Neuro‐ophthalmological examinations included best‐corrected visual acuity, slit lamp examination, fundoscopic examination, ocular motility examination, pupillary examination and a colour vision Ishihara test. In addition, optical coherence tomography (OCT) of the macula and optic nerve head using Heidelberg Spectralis (Heidelberg Engineering, Heidelberg, Germany) spectral domain OCT was done. OCT scans were reviewed for pathological changes in the macular region or at the optic disc, such as artery occlusion, optic disc oedema or atrophy and signs of focal retinal ischaemia like paracentral acute middle maculopathy (PAMM). PAMM was seen on macular OCT as a hyperreflective band within the inner nuclear layer (Klefter et al., 2025). Octopus (Haag‐Streit Group, Schweiz) 30‐degree visual field testing was included if possible. If automated perimetry was not possible, tangent screen or other types of visual field testing were performed. Finally, unilateral TAB was performed, aiming for surgery within 7–10 days after starting on glucocorticoids.

### 2.4 Biochemical measurements

Baseline blood samples were analysed for the levels of C‐reactive protein (CRP, VITROS®4600/5600, Ortho Clinical Diagnostics, New Jersey, USA), erythrocyte sedimentation rate (ESR, VACUETTE®SRS20/11, Greiner Bio‐One GmbH, Austria) and complete blood count, including platelet count (SYSMEX®XN‐9000, Sysmex Europe GmbH, Germany).

### 2.5 Ultrasound

Ultrasound of the temporal, facial, carotid and axillary arteries was performed bilaterally by three rheumatologists who have previously demonstrated high inter‐and intra‐observer reliability (Chrysidis et al., 2020). Ultrasound was done preferably within 1–3 days of referral and, when possible, prior to glucocorticoid administration. We used a GE Logiq® E10 (Milwaukee, Wisconsin, USA) ultrasound machine with a linear array 4–20 MHz and a 6–24 MHz hockey‐stick transducer. Patients were scanned in the supine position by an expert in rheumatology and ultrasonography. A non‐compressible artery (positive ‘compression sign’) and/or a ‘halo sign’ (a dark hypoechoic wall swelling around the artery lumen due to inflammatory wall thickening) in one or more arteries was considered indicative of GCA on ultrasound (Chrysidis et al., 2018; Schmidt, 2018; Schmidt et al., 1997). The ultrasound was classified as positive, negative or inconclusive for GCA.

### 2.6 2‐deoxy‐2‐[ ^18^F]fluoro‐D‐glucose positron emission tomography/computed tomography examination

2‐[^18^F]FDG PET/CT was performed from scalp to mid‐thigh with the arms positioned along the body (Siemens Biograph 64 PET/CT system, Siemens Healthcare, Erlangen, Germany). 2‐[^18^F]FDG PET/CT was ideally done within 3 days of administration of glucocorticoid. Patients were required to fast for 4 h prior to the tracer injection. Blood glucose was obtained, and 3 MBq kg^−1^ (1 MBq = 2703 × 10^−5^ Ci) 2‐[^18^F]FDG was administered intravenously. PET acquisition time was 3 min per bed position. A low‐dose enhanced CT, performed for anatomical mapping and attenuation correction, followed. PET attenuation‐corrected images were reconstructed using 2 iterations and 21 subsets, with time of flight and corrections for point spread function, attenuation and scatter. Using the scanner's software (Siemens Syngo VG80), images were reconstructed onto a 440 × 440 matrix and filtered with a 2.5 mm Gaussian kernel. A visual evaluation of the 2‐[^18^F]FDG uptake in the cranial arteries and the vessel wall of the large arteries was performed. Regarding cranial, carotid and vertebral arteries, a diffuse 2‐[^18^F]FDG uptake ≥ blood pool activity was considered positive for GCA. Whereas a diffuse 2‐[^18^F]FDG uptake ≥ liver activity in the rest of the vessel wall of the large arteries was regarded as positive for GCA (Lambæk et al., 2023; Nielsen et al., 2019; Nienhuis et al., 2020). The 2‐[^18^F]FDG PET/CT was considered positive for GCA if any artery showed signs of diffuse increased tracer uptake (Lambæk et al., 2023). If a scan was not suitable for grading due to a low target‐background ratio in the case of elevated blood glucose levels, it was considered inconclusive.

The 2‐[^18^F]FDG PET/CT scans were evaluated by experts in the field of clinical physiology and nuclear medicine who classified individual scans as positive, negative or inconclusive for GCA.

### 2.7 Temporal artery biopsy

Unilateral TAB was performed preferentially on the symptomatic side within 1 week of glucocorticoid administration. TAB was performed in accordance with hospital guidelines. Tissue samples were evaluated by experienced pathologists at the Department of Pathology, Rigshospitalet, Denmark, who were masked to the results of the other tests. The TAB was classified as positive biopsy, negative biopsy or inconclusive biopsy. A positive biopsy was defined by a transmural inflammation in the vessel wall, with or without giant cells (Tissue Pathways for Cardiovascular Pathology, 2018).

If a particular test was not performed, it was considered non‐available. Inconclusive test results occurred if a test was performed, but it could not be classified with certainty as positive or negative.

### 2.8 Final clinical and ophthalmological diagnosis and classification of giant cell arteritis

The final clinical diagnosis, definite GCA, definite not‐GCA or inconclusive, was based on clinical diagnosis at 6 months after baseline, a standard practice. Dividing GCA into cranial, large vessel or a combination of both was done by clinical experts in rheumatology in accordance with definitions seen in File S1. Differences were decided in consensus among the experts.

The final ophthalmological diagnosis was given by an expert in neuro‐ophthalmology based on the ophthalmological and neuro‐ophthalmological examinations at 6 months after baseline.

### 2.9 Data analysis and statistics

Demographics and baseline characteristics were used to describe the cohort. Q–Q plots and histograms were used to visualize data for normality. Categorical (qualitative) data were outlined using numbers and percentages and compared using a chi‐squared test. Normally distributed quantitative data were summarized using mean, range and standard deviation (SD) and compared using a *t*‐test. If data were not normally distributed, data were summarized using median and interquartile range (IQR) and compared using a Mann–Whitney *U* test.

Data in 2 × 2 tables were compared using a chi‐squared test and adjusted for multiple testing using the Bonferroni correction. For each diagnostic test, sensitivity (SN), specificity (SP), area under the curve (AUC) of the receiver operating characteristic (ROC) curve, positive predictive value (PPV) and negative predictive value (NPV) were given. The 95% confidence interval was calculated. Accuracy, positive likelihood ratio (PLR) and negative likelihood ratio (NLR) were calculated. The diagnostic odds ratio (DOR) was calculated for each test. A *p‐*value under 0.05 was considered statistically significant. The AUC was calculated and plotted in IBM SPSS Statistics. The statistical significance of differences between AUC values was calculated. The total sample size was calculated to be 102 patients.

## 3 RESULTS

### 3.1 Participant characteristics

**FIGURE 1:**
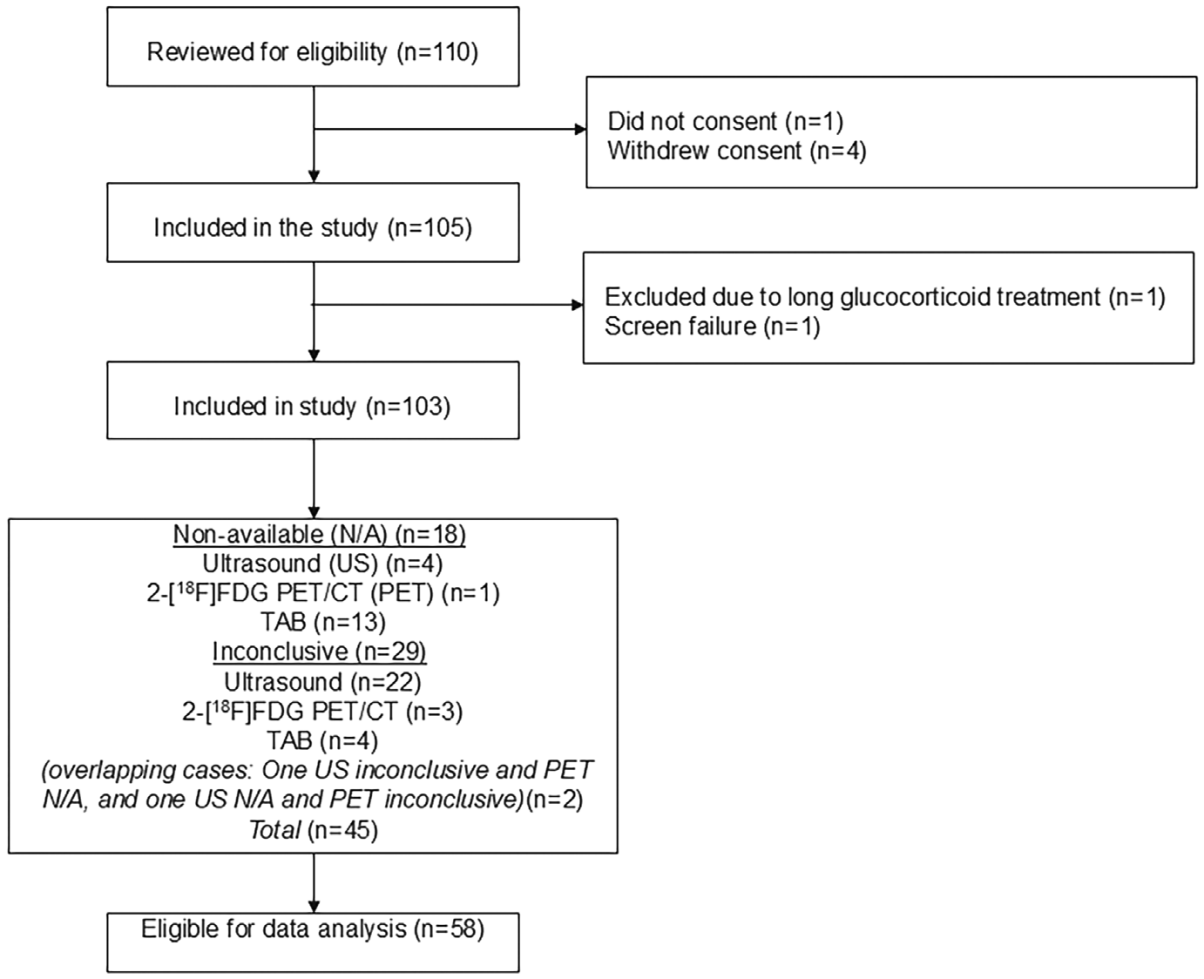
Study flowchart for participants.

A total of 110 patients were considered for inclusion. One patient did not consent, while four patients withdrew consent before completing study procedures. In total, 105 were included. Of these, 1 was excluded due to too long a treatment with glucocorticoid, and 1 was classified as a screen failure that was screened but not included in data analysis. A total of 103 patients were thus eligible for data analysis. When adjusted for non‐available and inconclusive tests, 58 patients were available for direct comparison of all three diagnostic tests (Figure 1). Non‐adjusted data are presented in Table 4.

Of the 103 patients, 61 (59%) received the final diagnosis of GCA, whereas 39 (38%) did not have GCA, and 3 (3%) were inconclusive. The age and sex distribution were similar in GCA and non‐GCA patients (Table 1). Six months after the final patient was included, 10 of the 103 patients (10%) had died.

**TABLE 1:** Patient characteristics.

	GCA (*n* = 61)	Non‐GCA (*n* = 39)	*p* value	Inconclusive (*n* = 3)	Total (*n* = 103)
Age (years), mean ± SD (range min–max)	75 ± 7.8 (54–87)	73 ± 9.4 (53–89)	0.295^a^	81.3 ± 12.4 (67–89)	74.4 ± 8.6 (53–89)
Females	39 (64)	27 (69)	0.587^b^	3 (100)	69 (67)
Males	22 (36)	12 (31)		0 (0)	34 (33)
1990 ACR criteria, *n* (%)					
≥3	51 (85)	17 (47)	0.0001^b^	2 (67)	70 (71)
<3	9 (15)	19 (53)		1 (33)	29 (29)
Time from glucocorticoid to test, median number of days (Q1–Q3); *n*					
Ultrasound	3 (1–4); 59	3 (0–6); 33	0.934^c^	2 (1–5); 3	3 (1–5); 95
Non‐available	2	6		0	8
2‐[^18^F]FDG PET/CT	4 (3–7); 59	4 (2–6); 33	0.425^c^	6 (5–7); 3	4 (2–7); 95
Non‐available	2	6		0	8
Temporal artery biopsy	7 (5–8); 53	7 (4–8); 28	0.948^c^	7 (6–8); 3	7 (5–8); 84
Non‐available	8	11		0	19
C‐reactive protein	0 (0–1); 60	0 (0–0); 33	0.162^c^	0 (0–1); 3	0 (0–0); 96
Non‐available	1	6		0	7
Erythrocyte sedimentation rate	0 (0–1); 51	0 (0–0); 32	0.061^c^	0 (0–1); 3	0 (0–0); 86
Non‐available	10	7		0	17
Platelet count	0 (0–1); 60	0 (0–0); 32	0.168^c^	0 (0–0); 2	0 (0–0); 94
Non‐available	1	7		1	9
GCA Subtypes					
Large vessel	4 (7)	0 (0)	0.177^b^	0 (0)	4 (5)
No large vessel	53 (93)	25 (100)		2 (100)	80 (95)
Cranial	14 (25)	1 (4)^d^	0.028^b^	0 (0)	15 (18)
No Cranial	43 (75)	24 (96)		2 (100)	69 (82)
Large vessel and cranial	38 (67)	0 (0)	<0.0001^b^	0 (0)	38 (45)
No Large vessel and cranial	19 (33)	25 (100)		2 (100)	46 (55)
PMR	0 (0)	2 (8)	0.032^b^	0 (0)	2 (2)
No PMR	57 (100)	23 (92)		2 (100)	82 (98)
Other diagnosis^e^	0* (0)	22 (88)	<0.001^b^	2 (100)	24 (29)
No other diagnosis	56 (100)	3 (12)		0 (0)	59 (71)
Dead, *n* (%)	4 (7)	3 (8)	0.829^b^	3 (100)	10 (10)
Alive, *n* (%)	57 (93)	36 (92)		0 (0)	93 (90)

	Demographics for patients with one or more non‐available or inconclusive test results				
	GCA (*n* = 23)	Non‐GCA (*n* = 19)	*p* value	Inconclusive (*n* = 3)	Total (*n* = 45)
Age (years), mean ± SD (range min–max)	74 ± 8.5 (54–86)	74 ± 9.5 (53–89)	0.952^a^	81 ± 12.4 (67–89)	75 ± 9.1 (53–89)
Females	13 (57)	13 (68)	0.435^b^	3 (100)	29 (64)
Males	10 (43)	6 (32)		0 (0)	16 (36)
1990 ACR criteria, *n* (%)					
≥3	17 (74)	8 (42)	0.084^b^	2 (67)	27 (60)
<3	5 (22)	8 (42)		1 (33)	14 (31)
Non‐available	1 (4)	3 (16)		0 (0)	4 (9)
Time from glucocorticoid to test, median number of days (Q1–Q3); *n*					
Ultrasound	1 (1–4); 21	4 (0–7); 15	0.184^c^	2 (1–5); 3	2 (1–5); 39
Non‐available	2	4		0	6
2‐[^18^F]FDG PET/CT	5 (3–7); 21	4 (2–6); 15	0.337^c^	6 (5–7); 3	5 (3–7); 39
Non‐available	2	4		0	6
Temporal artery biopsy	7 (5–8); 15	8 (6–8); 10	0.254^c^	7 (6–8); 3	7 (5–8); 28
Non‐available	8	9		0	17
C‐reactive protein	0 (0–1); 22	0 (0–0); 15	0.150^c^	0 (0–1); 3	0 (0–0); 40
Non‐available	1	4		0	5
Erythrocyte sedimentation rate	1 (0–2); 15	0 (0–0); 15	0.016^c^	0 (0–1); 3	0 (0–0); 33
Non‐available	8	4		0	12
Platelet count	0 (0–1); 22	0 (0–0); 14	0.230^c^	0 (0–0); 2	0 (0–0); 38
Non‐available	1	5		1	7
GCA Subtypes					
Large vessel	3 (15)	0 (0)	0.135^b^	0 (0)	3 (8)
No large vessel	17 (85)	14 (100)		2 (100)	33 (92)
Cranial	7 (35)	0 (0)	0.014^b^	0 (0)	7 (19)
No Cranial	13 (65)	14 (100)		2 (100)	29 (81)
Large vessel and cranial	10 (50)	0 (0)	0.002^b^	0 (0)	10 (28)
No Large vessel and cranial	10 (50)	14 (100)		2 (100)	26 (72)
PMR	0 (0)	2 (14)	0.086^b^	0 (0)	2 (6)
No PMR	20 (100)	12 (86)		2 (100)	34 (94)
Other diagnosis^e^	0 (0)	12 (86)	<0.0001^b^	2 (100)	14 (39)
No other diagnosis	20 (100)	2 (14)		0 (0)	22 (61)
Dead, *n* (%)	1 (4)	3 (16)	0.214^b^	3 (100)	7 (16)
Alive, *n* (%)	22 (96)	16 (84)		0 (0)	38 (84)

*Note*: A *p* value < 0.05 is statistically significant. 1990 ACR criteria = GCA classification criteria (not diagnostic) is a scoring system of five items (1) Age ≥ 50 years; (2) New onset headache; (3) Temporal artery abnormality; (4) Erythrocyte sedimentation rate ≥ 50 mm/h; (5) Abnormal artery biopsy. The lower part of the table presents demographics for patients with one or more non‐available or inconclusive test results (i.e. ultrasound, temporal artery biopsy and/or 2‐[^18^F]FDG PET/CT).

Abbreviations: GCA, giant cell arteritis; max, maximum; min, minimum; *n*, number of patients; PMR, polymyalgia rheumatica.

^a^ *T*‐test is used for comparing age.

^b^ Categoric data, gender, 1990 American College of Rheumatology (ACR) criteria and classification, are compared using the chi‐square test (*χ* ^2^‐test).

^c^ Mann–Whitney *U* Test is used for comparing the time from the start of the glucocorticoid to the performance of the individual test.

^d^ Patient with ultrasound positive, temporal artery biopsy and 2‐[^18^F]FDG PET/CT negative = false positive ultrasound.

^e^ Other diagnosis is given in the File S4 (e.g. infections, autoimmune diseases like rheumatoid arthritis, headache and ischaemic diseases).

The interval between commencement of glucocorticoid and the performance of ultrasound, 2‐[^18^F]FDG PET/CT and TAB, respectively, was 3 (Q1–Q3: 1–4 days), 4 (Q1–Q3: 3–7 days) and 7 (Q1–Q3: 5–8 days) days in the GCA group and 3 (Q1–Q3: 0–6 days) (*p* = 0.934, Mann–Whitney *U* test), 4 (Q1–Q3: 2–6 days) (*p* = 0.425, Mann–Whitney *U* test) and 7 (Q1–Q3: 4–8 days) (*p* = 0.948, Mann–Whitney *U* test) in the non‐GCA group (Table 1).

Of the 61 GCA patients, 57 could be subtyped. The majority had combined large vessel and cranial artery involvement (67%), followed by isolated cranial GCA (25%) and isolated large vessel GCA (7%).

### 3.2 Neuro‐ophthalmological findings

Overall, 68% of patients had ocular symptoms or findings, with equal distributions in the GCA and non‐GCA groups (*p* = 0.706, chi‐square test; Table 2). The most prevalent specific finding was arteritic anterior ischaemic optic neuropathy (A‐AION) in 11 patients. The largest subgroup overall was the non‐specific collective classification of ‘other diagnosis/signs’, which included entities such as central retinal artery occlusion, visual field defects, blurred vision and visual scintillation. In addition, transient diplopia was noted in four patients in the GCA group and one patient in the non‐GCA group.

**TABLE 2:** Final neuro‐ophthalmological diagnosis.

	GCA (*n* = 61)	Non‐GCA (*n* = 39)	*p* value	Inconclusive (*n* = 3)	Total (*n* = 103)
Ocular involvement, *n* (%)	40 (66)	27 (69)	0.706^a^	3 (100)	70 (68)
A‐AION	11 (18)	0 (0)	N/A^b^	1^c^ (33)	12 (12)
A‐PION	0 (0)	0 (0)	N/A	0 (0)	0 (0)
NA‐AION	0 (0)	3 (8)	N/A	0 (0)	3 (3)
NA‐PION	0 (0)	1 (3)	N/A	0 (0)	1 (1)
Oculomotor nerve palsy	0 (0)	0 (0)	N/A	0 (0)	0 (0)
Trochlear nerve palsy	0 (0)	0 (0)	N/A	0 (0)	0 (0)
Abducens nerve palsy	0 (0)	0 (0)	N/A	0 (0)	0 (0)
Other diagnosis/signs^d^	29 (48)	23 (59)	0.503^a^	2 (67)	54 (52)
Vision loss	1	0		1	2
Visual disturbance	19	10		1	29
Transient visual loss	3	5		0	8
Diplopia	4	2		0	7
Visual field defects	0	2		0	2
Artery occlusions/Ocular pain	2	2		0	4
Optic neuropathy	0	2		0	2
No ocular involvement	21 (34)	12 (31)	N/A	0 (0)	33 (32)

*Note*: One patient was deemed AION and later classified as NA‐AION due to non‐GCA final diagnosis.

Abbreviations: A‐AION, arteritic anterior ischaemic optic neuropathy; AION, anterior ischaemic optic neuropathy; A‐PION, arteritic posterior ischaemic optic neuropathy; AUC, area under the curve; GCA, giant cell arteritis; *n*, number of patients; N/A, non‐available; NA‐AION, non‐arteritic anterior ischaemic optic neuropathy; NA‐PION, non‐arteritic posterior ischaemic optic neuropathy.

^a^ Chi‐square test.

^b^ A‐AION can only be present in the setting of GCA.

^c^ In this patient, the final neuro‐ophthalmological diagnosis was AION, and the final rheumatological diagnosis was deemed inconclusive.

^d^ The specific other diagnosis is provided in the File S5.

### 3.3 Blood tests

Compared with the non‐GCA group, patients with GCA had a higher median CRP of 50.0 mg/L (IQR: 10.0–90.0 mg/L) vs. 5.5 mg/L (IQR: 1.4–20.3 mg/L), a higher ESR of 53.0 mm (IQR: 37.0–85.5 mm) vs. 15.0 mm (IQR: 8.8–27.0 mm) and a higher platelet count of 364.0 10^9^/L (IQR: 306.5–428.0 10^9^/L) vs. 267.5 10^9^/L (IQR: 213.5–294.0 10^9^/L) (all *p* < 0.001, Mann–Whitney *U* test) (File S2).

### 3.4 Comparative performance of ultrasound, 2‐[ ^18^F]FDG PET/CT and TAB for diagnosing GCA

The direct comparison of the three diagnostic tests was performed in the subgroup of 58 patients, who had complete data on positive or negative test results, that is, where patients with any non‐available or inconclusive tests had been excluded (Table 3). Ultrasound had the highest sensitivity of 82% (95% CI: 66%–92%) and a specificity of 90% (95% CI: 68%–99%), resulting in a PPV of 94% (95% CI: 81%–98%) and a NPV of 72% (95% CI: 57%–84%). By comparison, 2‐[^18^F]FDG PET/CT and TAB both achieved a higher specificity of 100% (both 95% CI: 83%–100%), but with lower sensitivities of 74% (95% CI: 57%–87%) and 68% (95% CI: 51%–83%), respectively. Correspondingly, the PPV is higher for 2‐[^18^F]FDG PET/CT and the NPVs are lower than for ultrasound (Table 3). While the overall accuracy was numerically the highest for ultrasound at 85% (73%–93%) and the diagnostic odds ratio numerically the highest for 2‐[^18^F]FDG PET/CT at 111.29 (6.16–2009.04), the AUC values of 0.858 (0.752–0.964), 0.868 (0.777–0.960) and 0.842 (0.742–0.942), respectively, showed comparable diagnostic performance (*p* = 0.708 to 0.889). Test results including inconclusive and non‐available values are shown in Table 4. Ultrasound had the largest proportion of inconclusive tests (21%), followed by Table (4%) and 2‐[^18^F]FDG PET/CT (3%). TAB had the largest proportion of non‐available tests (13%), followed by ultrasound (4%) and 2‐[^18^F]FDG PET/CT (1%), Table 4. The non‐available values were equally distributed in the GCA and non‐GCA groups (*p =* 0.573, chi‐squared test). Of the 13 non‐available TAB samples, 5 patients had undergone surgery, but the artery could not be localized clinically for biopsy sampling.

**TABLE 3:** Diagnostic performance of temporal artery biopsy, 2‐[^18^F]FDG PET/CT and ultrasound in patients with complete positive and negative results for all three tests.

	GCA (*n* = 38)	Non‐GCA (*n* = 20)	Total (*n* = 58)	Performance, % (95% CI)
Ultrasound, *n*				Sensitivity: 82% (66%–92%) Specificity: 90% (68%–99%) AUC: 0.858 (0.752–0.964) PPV: 94% (81%–98%) NPV: 72% (57%–84%) Accuracy: 85% (73%–93%) PLR: 8.16 (2.17–30.64) NLR: 0.20 (0.10–0.41) DOR: 39.86 (7.46–212.87)
Positive	31	2	33	
Negative	7	18	25	
2‐[^18^F]FDG PET/CT, *n*				Sensitivity: 74% (57%–87%) Specificity: 100% (83%–100%) AUC: 0.868 (0.777–0.960) PPV: 100% (88%–100%) NPV: 67% (54%–77%) Accuracy: 83% (71%–91%) PLR: – NLR: 0.26 (0.15–0.45) DOR^a^: 111.29 (6.16–2009.04)
Positive	28	0	28	
Negative	10	20	30	
TAB, *n*				Sensitivity: 68% (51%–83%) Specificity: 100% (83%–100%) AUC: 0.842 (0.742–0.942) PPV: 100% (87%–100%) NPV: 63% (51%–73%) Accuracy: 79% (67%–89%) PLR: – NLR: 0.32 (0.20–0.50) DOR^a^: 86.92 (4.86–1556.09)
Positive	26	0	26	
Negative	12	20	32	

*Note*: AUC: TAB vs. 2‐[^18^F]FDG PET/CT (*p* = 0.708), AUC: TAB vs. ultrasound (*p* = 0.829), AUC: ultrasound vs. 2‐[^18^F]FDG PET/CT (*p* = 0.889), File S6.

Abbreviations: AUC, area under the curve; CI, confidence interval; DOR, diagnostic odds ratio; GCA, giant cell arteritis; *n*, number of patients; NLR, negative likelihood ratio; NPV, negative predictive value; PLR, positive likelihood ratio; PPV, positive predictive value.

^a^ Haldane Correction.

**TABLE 4:** Diagnostic performance of temporal artery biopsy, ultrasound and ^18^F‐FDG/PET in entire cohort.

	GCA (*n* = 61)	Non‐GCA (*n* = 39)	Inconclusive (*n* = 3)	Total (*n* = 103)	Performance, % (95% CI)
Ultrasound, *n*	60	36	3	99	Sensitivity: 78% (65%–89%) Specificity: 92% (75%–99%) AUC: 0.858 (0.752–0.964) PPV: 95% (84%–99%) NPV: 69% (56%–79%) Accuracy: 83% (73%–91%) PLR: 10.20 (2.67–38.91) NLR: 0.23 (0.14–0.40) DOR: 43.64 (8.90–213.85)
Positive	40	2	0	42	
Negative	11	24	0	35	
Inconclusive	9	10	3	22	
Non‐available	1	3	0	4	
2‐[^18^F]FDG PET/CT, *n*	60	39	3	102	Sensitivity: 69% (56%–81%) Specificity: 100% (91%–100%) AUC: 0.868 (0.777–0.960) PPV: 100% (91%–100%) NPV: 68% (59%–76%) Accuracy: 81% (72%–89%) PLR: – NLR: 0.31 (0.21–0.46) DOR^b^: 168.57 (9.81–2895.28)
Positive	40	0	0	40	
Negative	18	38	3	59	
Inconclusive	2	1	0	3^a^	
Non‐available	1	0	0	1	
Temporal artery biopsy, *n*	54	33	3	90	Sensitivity: 58% (43%–72%) Specificity: 100% (89%–100%) AUC: 0.842 (0.742–0.942) PPV: 100% (88%–100%) NPV: 61% (53%–69%) Accuracy: 75% (64%–84%) PLR: – NLR: 0.42 (0.30–0.58) DOR^b^: 91.93 (5.33–1584.89)
Positive	29	0	1	30	
Negative	21	33	2	56	
Inconclusive	4	0	0	4	
Non‐available	7	6	0	13	

*Note*: Sensitivity and specificity of all three tests, taking inconclusive tests into account and adding inconclusive results to positive and negative results. Ultrasound, adding inconclusive results to positive results, had a sensitivity of 82% (95% CI: 70%–91%) and a specificity of 67% (95% CI: 49%–81%). Ultrasound, adding inconclusive results to negative results, had a sensitivity of 67% (95% CI: 53%–78%). Adding inconclusive results to positive 2‐[^18^F]FDG PET/CT had a sensitivity of 70% (95% CI: 57%–81%) and a specificity of 97% (87%–100%). Adding inconclusive results to negative 2‐[^18^F]FDG PET/CT had a sensitivity of 67% (95% CI: 53%–78%) and a specificity of 100% (91%–100%). Adding inconclusive results to positive temporal artery biopsy had a sensitivity of 61% (95% CI: 47%–74%) and a specificity of 100% (95% CI: 89%–100%). Adding inconclusive results to negative 2‐[^18^F]FDG PET/CT had a sensitivity of 54% (95% CI: 40%–67%) and a specificity of 100% (95% CI: 89%–100%).

Abbreviations: AUC, area under the curve; CI, confidence interval; DOR, diagnostic odds ratio; GCA, giant cell arteritis; *n*, number of patients; NLR, negative likelihood ratio; NPV, negative predictive value; PLR, positive likelihood ratio; PPV, positive predictive value.

^a^ 3 out of 3 had high blood sugar levels (range: 11.7–12.30 mmol/L) resulting in, low target‐to‐background ratio. AUC TAB vs. 2‐[^18^F]FDG PET/CT (*p* = 0.708), AUC TAB vs. ultrasound (*p* = 0.829), AUC ultrasound vs. 2‐[^18^F]FDG PET/CT (*p* = 0.889).

^b^ Haldane Correction.

### 3.5 Performance of TAB and 2‐[ ^18^F]FDG PET/CT in inconclusive and negative ultrasound tests

When ultrasound was inconclusive, 2‐[^18^F]‐FDG PET/CT had a higher sensitivity than TAB at 75% (95% CI: 35%–97%) vs. 38% (95% CI: 9%–76%) (*p* = 0.250), with the same specificity of 100% (both 95% CI: 69%–100%). Correspondingly, the NPV was numerically higher for 2‐[^18^F]‐FDG PET/CT than for TAB (Table 5). Based on the subgroup of 18 patients with complete data, the AUC was numerically higher for 2‐[^18^F]‐FDG PET/CT than for TAB, although it did not reach statistical significance (*p* = 0.258). The findings were similar when the ultrasound was negative (Table 6).

**TABLE 5:** Performance of temporal artery biopsy and 2‐[^18^F]FDG PET/CT when ultrasound was inconclusive.

	GCA (*n* = 8)	Non‐GCA (*n* = 10)	Total (*n* = 18)	Performance, % (95% CI)
2‐[^18^F]FDG PET/CT, *n*				Sensitivity: 75% (35%–97%) Specificity: 100% (69%–100%) AUC: 0.875 (0.685–1.065) PPV: 100% (54%–100%) NPV: 83% (60%–94%) Accuracy: 89% (65%–99%) PLR: – NLR: 0.25 (0.08–0.83) DOR^a^: 54.6 (2.25–1326.28)
Positive	6	0	6	
Negative	2	10	12	
TAB, *n*				Sensitivity: 38% (9%–76%) Specificity: 100% (69%–100%) AUC: 0.688 (0.425–0.950) PPV: 100% (29%–100%) NPV: 67% (54%–77%) Accuracy: 72% (47%–90%) PLR: – NLR: 0.62 (0.37–1.07) DOR^a^: 13.36 (0.58–308.04)
Positive	3	0	3	
Negative	5	10	15	

*Note*: AUC: TAB vs. 2‐[^18^F]FDG PET/CT (*p* = 0.258), File S7.

Abbreviations: AUC, area under the curve; CI, confidence interval; DOR, diagnostic odds ratio; GCA, giant cell arteritis; *n*, number of patients; NLR, negative likelihood ratio; NPV, negative predictive value; PLR, positive likelihood ratio; PPV, positive predictive value.

^a^ Haldane Correction.

**TABLE 6:** Performance of temporal artery biopsy and 2‐[^18^F]FDG PET/CT when ultrasound was negative.

	GCA (*n* = 7)	Non‐GCA (*n* = 18)	Total (*n* = 25)	Performance, % (95% CI)
18‐FDG PET/CT, *n*				Sensitivity: 29% (3.67%–71%) Specificity: 100% (82%–100%) AUC: 0.643 (0.374–0.911) PPV: 100% (16%–100%) NPV: 78% (69%–85%) Accuracy: 80% (59%–93%) PLR: – NLR: 0.71 (0.45–1.14) DOR^c^: 16.82 (0.698–405.29)
Positive	2	0	2	
Negative	5^a^	18	23	
TAB, *n*				Sensitivity: 14% (0.36–58) Specificity: 100% (82%–100%) AUC: 0.571 (0.304–0.839) PPV: 100% (2.5%–100%) NPV: 75% (69%–80%) Accuracy: 76% (55%–91%) PLR: – NLR: 0.86 (0.63–1.16) DOR^c^: 8.54 (0.308–236.91)
Positive	1	0	1	
Negative	6^b^	18	24	

*Note*: AUC: TAB vs. 2‐[^18^F]FDG PET/CT (*p* = 0.709), File S8.

Abbreviations: AUC, area under the curve; CI, confidence interval; DOR, diagnostic odds ratio; GCA, giant cell arteritis; *n*, number of patients; NLR, negative likelihood ratio; NPV, negative predictive value; PLR, positive likelihood ratio; PPV, positive predictive value.

^a^ A total of 4 out of 5 patients were both negative on TAB and 2‐[^18^F]FDG PET/CT. The last 2‐[^18^F]FDG PET/CT negative patient had a positive TAB.

^b^ A total of 5 out of 6 patients had classical clinical symptoms, and in some patients, elevated blood biomarkers. The last TAB negative patient had vertebral vasculitis on 2‐[^18^F]FDG PET/CT.

^c^ Haldane Correction.

### 3.6 Non‐GCA findings and adverse events

Findings on 2‐[^18^F]FDG PET/CT which could explain some of the symptoms mimicking GCA included sinusitis (*n* = 2), occipital infarction (*n* = 1), mandibular osteomyelitis (n = 1) and, retrospectively, a meningioma in the left temporal fossa media (n = 1). Incidental findings on 2‐[^18^F]FDG PET/CT scans included lung cancer (n = 1), a benign pulmonary infiltrate (n = 1), a benign osteolytic bone lesion in the scapula (n = 1), a benign duodenal tumour (n = 1) and a benign breast tumour (n = 1). No adverse events were noted in relation to the diagnostic testing.

### 3.7 Comparative performance of ultrasound, 2‐[ ^18^F]FDG PET/CT and TAB in A‐AION patients

Of the 12 A‐AION patients, 11 were diagnosed with GCA and 1 was diagnosed with A‐AION by a neuro‐ophthalmologist but was categorized as inconclusive by a rheumatologist (Table 2). Shown in the File S3 are the contingency tables between ultrasound, 2‐[^18^F]FDG PET/CT and TAB. Of important note, four patients with a positive TAB had a negative 2‐[^18^F]FDG PET/CT. Of these four, three had A‐AION. Of these three, two had PAMM.

## 4 DISCUSSION

This multimodal diagnostic cohort study demonstrated that ultrasound, 2‐[^18^F]FDG PET/CT and TAB all performed well in distinguishing between GCA and non‐GCA. While the overall diagnostic accuracies were comparable, ultrasound seemed to favour sensitivity, whereas the two other tests achieved perfect specificities with a mean of 100%. Inconclusive and non‐available test results were particularly prevalent for ultrasound, whereas the highest number of conclusive test results was achieved with 2‐[^18^F]FDG PET/CT. In the event of inconclusive ultrasound results, the overall accuracy of TAB and 2‐[^18^F]FDG PET/CT was comparable, although 2‐[^18^F]FDG PET/CT appeared to be more sensitive.

**FIGURE 2:**
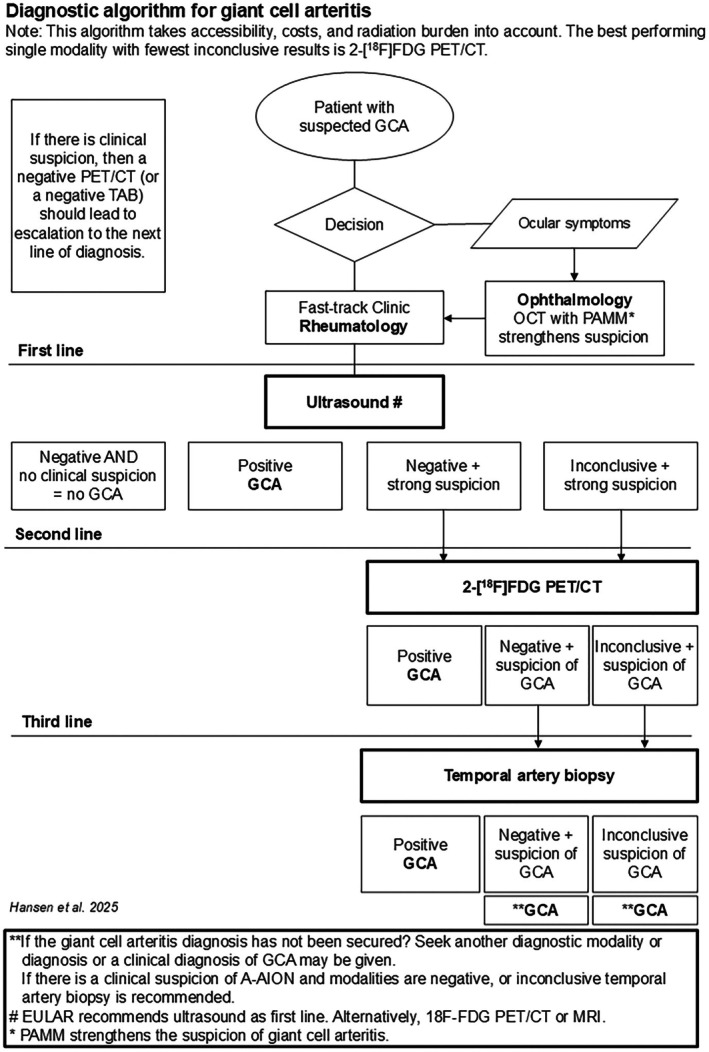
Diagnostic flowchart for giant cell arteritis. Algorithm for a multimodal diagnostic approach to giant cell arteritis. GCA = Giant cell arteritis; EULAR = European Alliance of Associations for Rheumatology; MRI = Magnetic Resonance Imaging; A‐AION = arteritic anterior ischaemic optic neuropathy; PAMM = Paracentral acute middle maculopathy; TAB = temporal artery biopsy; 2‐[^18^F]FDG PET/CT = 2‐deoxy‐2‐[^18^F]fluoro‐D‐glucose positron emission tomography/computed tomography.

The fact that no single test can confirm or rule out GCA with 100% accuracy supports the recommendation by EULAR to apply a multimodal approach in a fast‐track setup with imaging as first‐line testing (Dejaco et al., 2024). Because overall diagnostic accuracies were comparable, other characteristics of the modalities may be reflected upon when considering the order of testing. We suggest that 2‐[^18^F]FDG PET/CT may be a suitable second‐line test, especially if the initial ultrasound is inconclusive and the suspicion of GCA remains high. This would reserve TAB for third‐line testing (Figure 2). If a reasonable clinical suspicion of GCA remains, then a negative 2‐[^18^F]FDG PET/CT (or a negative TAB) should lead to escalation to the next line of diagnostic testing. This is important because false negatives were observed with 2‐[^18^F]FDG PET/CT or TAB. If several diagnostic tests for GCA have turned out negative, alternative differential diagnoses should be considered. Should GCA still remain the most probable entity, it may be considered as a clinical diagnosis. In the subpopulation of inconclusive ultrasound, 2‐[^18^F]FDG PET/CT had a sensitivity of 29%. While this appears low, the corresponding sensitivity for TAB was 14%. While the low numbers do not allow for meaningful statistical analysis, the overall pros and cons of these two tests lead us to conclude that 2‐[^18^F]FDG PET/CT may have an advantage as a second‐line test if ultrasound is inconclusive. The diagnostic performance of ultrasound in this study was comparable to or better than previously published results and confirmed its overall comparable accuracy with TAB (Hansen et al., 2023; Luqmani et al., 2016). The presence of a false positive ‘halo’ sign has been reported in up to 4.6% of patients and may contribute to the lower specificity. This finding can be due to other types of vasculitis (Fernández‐Fernández et al., 2020). The relatively high sensitivity of ultrasound may be associated with the inclusion of multiple arterial branches in the scan protocol rather than with the temporal arteries alone (Dejaco et al., 2024; Hop et al., 2020; Prearo et al., 2022).

The performance of 2‐[^18^F]FDG PET/CT reached a moderately high sensitivity and a very high specificity of 100%. This is in accordance with other studies (Sammel et al., 2019). Sammel et al. found a sensitivity of 71% (95% CI: 48%–89%) and a specificity of 91% (95% CI: 78%–97%). They found an NPV of 98% (95% CI: 87%–100%) and concluded that 2‐[^18^F]FDG PET/CT was a good test for ruling out GCA. Our study found a lower NPV of 67% (95% CI: 54%–77%). This difference could be influenced by glucocorticoid treatment as demonstrated by Lambæk et al., who compared PET/CT with TAB and found an overall 2‐[^18^F]FDG PET/CT sensitivity of 76% (95% CI: 63%–90%) and a specificity of 100% (95% CI: 92%–100%). The sensitivity was 85% (95% CI: 72%–99%) if glucocorticoid treatment was ≤3 days, but was considerably lower at 55% (95% CI: 25%–84%) if glucocorticoid treatment was >3 days (Lambæk et al., 2023). As the median time from glucocorticoid to 2‐[^18^F]FDG PET/CT was 4 days (IQR: 2–7 days), glucocorticoid could have reduced the sensitivity of 2‐[^18^F]FDG PET/CT in our study.

The sensitivity and specificity of TAB were on par with other studies (Hansen et al., 2023). The sensitivity generally varies from 39% to 77% (Luqmani et al., 2016; Rubenstein et al., 2020). The sensitivity of TAB has been calculated in a Bayesian analysis to be 87.1% (95% CI: 81.8%–91.7%) in one study (Niederkohr & Levin, 2007). The lower sensitivity can be due to skip lesions, the histopathological interpretation or treatment with glucocorticoids (Luqmani et al., 2016). The median time from glucocorticoid to TAB was 7 days. Studies have found that a longer duration of glucocorticoid can reduce the sensitivity. Specifically, Papadakos et al. found that glucocorticoid treatment for more than 7 days reduced the proportion of positive TABs (Papadakos et al., 2023). While the influence of specimen length is debated, we aimed for 1–2 cm specimens in agreement with hospital guidelines to accommodate for skip lesions. Of note, skip lesions are rare, reported to be 8.5% of TAB in active vasculitis cases (Poller et al., 2000). To reduce false negative histopathological interpretation of TAB, only a few expert pathologists handled the specimens.

That A‐AION was one of the most frequent neuro‐ophthalmological findings is in accordance with other studies (Bilton & Mollan, 2023; Hansen et al., 2023). It is noteworthy that of the four patients with positive TAB and a negative 2‐[^18^F]FDG PET/CT, three cases were diagnosed with A‐AION, and two of the three patients had PAMM on OCT (three out of four had a positive ultrasound), which has previously been found to be an OCT biomarker of GCA and A‐AION (Klefter et al., 2025; Mairot et al., 2022). All four patients were given the final diagnosis of GCA. Possible explanations for why 2‐[^18^F]FDG PET/CT could not detect GCA in these patients could be glucocorticoid treatment or potentially low levels of inflammation with a correspondingly low FDG uptake. Another explanation of this finding is a lower sensitivity of cranial GCA (Rottenburger et al., 2021). Of note, all four patients had elevated CRP and ESR.

An important observation was the prevalence of inconclusive and non‐available test results. One in five ultrasound examinations was inconclusive, and this was considerably higher than for TAB and 2‐[^18^F]FDG PET/CT. Several factors may be considered. First, ultrasound is an operator‐dependent technique that, in our study, sought to be eliminated using expert sonographers and standardized procedures (Schmidt & Schäfer, 2024) and previously demonstrated high inter‐observer agreement (Chrysidis et al., 2018). Second, in the study, the ultrasound exams were deemed inconclusive in relation to the diagnosis if the findings were normal, but the steroid treatment was more than 3 days. Although ultrasound was prioritized in our fast‐track setting, it is possible that glucocorticoid treatment could have influenced the findings in a few patients. However, only a short duration of glucocorticoid treatment before ultrasound was seen in the study. From a clinical point of view, it may be argued that admitting inconclusive results may improve patient safety in comparison to forcing a choice in uncertain cases. This approach may avoid erroneous diagnoses and prompt additional testing.

Of the three 2‐[^18^F]FDG PET/CT scans that were inconclusive, two patients had elevated blood glucose levels, reducing the target‐to‐background ratio. TAB was inconclusive in a similarly low number of patients. However, TAB had the highest number of non‐available cases, representing 12.6%. In the majority (*n* = 8), patients had declined the procedure, possibly reflecting a preference for non‐invasive examinations. In addition, five TAB cases were non‐available because the artery could not be localized clinically for biopsy sampling, reflecting the technical challenges of the procedure. The few non‐available results for the two other modalities most likely reflect individual logistical challenges that may be difficult to completely avoid.

The high sensitivity and availability, low cost, non‐invasive nature and immediate real‐time sonographic expert diagnosis make ultrasound suitable as the first‐line diagnostics of suspected GCA. This is in accordance with EULAR recommendations (Dejaco et al., 2024; Hellmich et al., 2020). Ultrasound was the test with the highest number of inconclusive results, but it was also the fastest and there was no diagnostic delay. However, the need for highly trained ultrasonographers and the high proportion of inconclusive ultrasound tests mean that one or more additional test modalities should be readily available. Implementing a fast‐track setting will typically involve centralizing the work‐up to achieve a high level of operator experience as recommended by EULAR (Dejaco et al., 2024). Although the proportion of inconclusive tests might be considered a potential barrier for the implementation, recognizing an uncertain result avoids the consequences of a potentially erroneous dichotomous forced choice. This enables immediate referral for second‐line testing. Notably, a conclusive result could be obtained in four out of five patients using a non‐invasive modality. Traditionally, TAB has been considered the gold standard test for diagnosing GCA, and it should therefore be expected to already be available in most centres that specialize in vasculitis and in neuro‐ophthalmology. The cost is higher than for ultrasound (Hansen et al., 2023; Schmidt & Schäfer, 2024), and our results indicated that TAB might have a lower sensitivity than 2‐[^18^F]FDG PET/CT if the preceding ultrasound was inconclusive or negative, although it might have higher sensitivity in the particular case of A‐AION. TAB is an invasive procedure, which may influence patient preferences if comparable non‐invasive diagnostic options are available. Finally, the histopathological diagnosis may become available 1–2 weeks after the biopsy sampling, and a diagnostic delay may be shortened with non‐invasive imaging modalities. The lowest number of non‐available and inconclusive results was found for 2‐[^18^F]FDG PET/CT. 2‐[^18^F]FDG PET/CT had the additional advantages over TAB and, to a lesser extent, ultrasound that it is able to detect vasculitis in a much larger anatomical area, including in the aorta and the vertebral arteries. Therefore, 2‐[^18^F]FDG PET/CT may be of important value in helping to subtype GCA and provide information about relevant differential diagnoses. Although 2‐[^18^F]FDG PET/CT incurs an exposure to radioactive radiation of 6–8 mSv, depending on body weight, which is 2 times the average annual background exposure for a Danish citizen (The Danish Health Authority, 2024). The radiation burden is accepted as safe in routine clinical imaging. However, this modality is considered more expensive than ultrasound (Schmidt & Schäfer, 2024). In general, the accessibility to PET/CT scanners is lower than for ultrasound and TAB, although accessibility to 2‐[^18^F]FDG PET/CT in Denmark is high (Eurostat, 2024).

The 2021 ACR guidelines made a conditional recommendation to prioritize TAB over ultrasound in the work‐up of suspected GCA (Maz et al., 2021). However, newer guidelines by EULAR have recommended imaging (ultrasound or PET/CT) as the first‐line diagnostic (Dejaco et al., 2024). The revised classification criteria for GCA, published jointly by the ACR and EULAR, apply equal weighting to a positive ultrasound and TAB result (Ponte et al., 2022). There appears to be increasing evidence that imaging should be considered on par with and possibly before TAB, which is in line with our suggestion.

Taking diagnostic performance, inconclusive results, costs, invasiveness, radiation exposure and accessibility into consideration, we propose a clinical flowchart (Figure 2) for fast‐track multimodal diagnosis of GCA. It applies to settings similar to those already found in Denmark with good accessibility to 2‐[^18^F]FDG PET/CT. A prerequisite for our proposed GCA diagnostic clinical flowchart is a fast, accessible and multimodal approach with minimal diagnostic delay, preferably within 3–7 days.

We noted that in this cohort of 103 patients, predominantly included from the eye department, no GCA patients had concomitant polymyalgia rheumatica (PMR) and only 2 in the non‐GCA group had PMR. The few PMR patients could be due to selection bias due to inclusion from the Department of Ophthalmology. This could indicate that our study cohort was dominated by cranial and ischaemic phenotypes as defined in the GCA‐PMR spectrum disease (Tomelleri et al., 2023). Our findings indicate that PMR is rarely seen in GCA patients which predominantly presents at the Department of Ophthalmology and that the cranial and ischaemic phenotypes rarely have PMR.

Strengths of the study include consecutive sampling of a large cohort from a single centre, with all patients undergoing the same comprehensive examination process including ultrasound, 2‐[^18^F]FDG PET/CT and TAB, as well as a neuro‐ophthalmological examination, allowing for direct comparison.

One limitation is a possible selection bias due to the inclusion of the majority of patients from the Department of Ophthalmology and a highly specialized Department of Rheumatology. This may explain the predominance of combined large vessel and cranial GCA with a low number of PMR cases as well as the large proportion of ocular symptoms and signs. Another limitation is the number of non‐available test results which, however, may reflect the actual clinical nature of the study. Additionally, the distribution of non‐available tests might suggest a patient preference for non‐invasive testing. Of the 103 eligible patients, only 58 patients were available for head‐to‐head comparison, and this may create selection bias, a limitation of the study. Randomization was not possible due to the acute nature of GCA. Due to the prospective nature of the study design, a concern could be patients lost to follow‐up or drop out, but only a few patients dropped out in our study. As a result of the setup involving three major clinical departments, the adjudicators were not blinded to the 2‐[^18^F]FDG PET/CT and TAB results. This is a limitation, as it potentially can inflate the specificity of the index tests. Finally, although the cohort was large, some of the analyses have suboptimal statistical power as shown by the wide confidence intervals, suggesting the need for additional, larger studies. Exclusion of patients with active cancer was chosen due to the difficulty in discriminating between paraneoplastic and large vessel forms of vasculitis. This choice may have resulted in selection bias and reduced generalizability.

In conclusion, ultrasound, 2‐[^18^F]FDG PET/CT and TAB were comparably accurate for diagnosing GCA. The sensitivity of 2‐[^18^F]FDG PET/CT was found to be on par with ultrasound. However, due to its higher availability and lower cost, the EULAR recommendation of using ultrasound as the first‐line test is supported. When ultrasound is inconclusive or negative, with a continued clinical suspicion of GCA, 2‐[^18^F]FDG PET/CT is suggested as the second‐line test, especially in settings where PET/CT is readily available. We suggest that TAB is reserved for third‐line testing, especially when the suspicion of GCA remains high and in cases with suspected A‐AION and concomitant PAMM.

## AUTHOR CONTRIBUTIONS

**Michael S. Hansen**: Literature review, study design, methodology, data collection, formal analysis, drafting of paper and funding acquisition. **Lene Terslev**: Rheumatology expert in GCA and ultrasound, methodology, data collection, draft and critical review of paper. **Uffe M. Døhn**: Rheumatology expert in GCA and ultrasound, methodology, data collection, draft and critical review of paper. **Viktoria Fana**: Rheumatology expert in GCA and ultrasound, methodology, data collection, draft and critical review of paper. **Jane M. Brittain**: Expert in GCA and PET methodology, data collection, draft and critical review of paper. **Oliver N. Klefter**: Methodology, interpretation of results, critical review of statistics and paper. **Yousif Subhi**: Methodology, interpretation of results, critical review of statistics and paper. **Steffen Heegaard**: Pathologist, study design, methodology, data collection, critical review of the paper, supervision and funding acquisition. **Mads R. Jensen**: Expert in GCA and PET, methodology, data collection, draft and critical review of paper. **Anne K. Wiencke**: Head of Ophthalmic Surgery Ward, Methodology, supervision and critical review of paper. **Steffen Hamann**: Neuro‐ophthalmologist expert in GCA, study design, methodology, data collection, drafting and critical review of the paper, supervision and funding acquisition.

## FUNDING INFORMATION

This research was funded by Synoptik‐Fonden and Gigtforeningen (The Danish Rheumatism Association) (Grants R218‐A7908 and R201‐A7279).

## CONFLICT OF INTEREST STATEMENT

None of the authors has any conflict of interest to disclose.

## DISCLOSURE

Lene Terslev: UCB, Novartis, Janssen for lecture on ultrasound in rheumatology.

## Supporting information


**File S1.** Definition of GCA subtypes.

**File S2.** Baseline biomarkers.

**File S3.** 2 × 2 Contingency tables.

**File S4.** Table of final specific clinical diagnosis at 6 months follow‐up classified as ‘other diagnosis’ by a rheumatologist in Table 1.

**File S5.** Table of specific final neuro‐ophthalmological ‘other diagnosis/signs’ in Table 2.

**File S6.** Receiver operating characteristic curve.

**File S7.** Receiver operating characteristic curve.

**File S8.** Receiver operating characteristic curve.
