# Supplementary material for: Ultrasound, PET/CT or temporal artery biopsy for giant cell arteritis? A prospective diagnostic accuracy study (the GAME‐study)
Source: Acta Ophthalmol. 2026 Mar 7;104(6):e695–708. doi: 10.1111/aos.70114 (PMC13460362; doi:10.1111/aos.70114)
Supplement: Supplementary file 1 [file AOS-104-e695-s001.docx]

**Supplementary file 8.** Receiver operating characteristic curve. Shown are the ROC curves for temporal artery biopsy (AUC 0.571) and 2-[^18^F]FDG PET/CT (AUC = 0.643) for patients with negative ultrasound. AUC = area under the curve. In red, reference line. AUC TAB vs. 2-[^18^F]FDG PET/CT (*p* = 709).
