# Supplementary material for: Ultrasound, PET/CT or temporal artery biopsy for giant cell arteritis? A prospective diagnostic accuracy study (the GAME‐study)
Source: Acta Ophthalmol. 2026 Mar 7;104(6):e695–708. doi: 10.1111/aos.70114 (PMC13460362; doi:10.1111/aos.70114)
Supplement: Supplementary file 2 [file AOS-104-e695-s002.docx]

**Supplementary file 6.** Receiver operating characteristic curve. Shown are the ROC curves for temporal artery biopsy (AUC 0.842), 2-[^18^F]FDG PET/CT (AUC = 0.868) and ultrasound (AUC = 0.858). AUC = area under the curve. In red, reference line. AUC: TAB vs. 2-[^18^F]FDG PET/CT (*p* = 0.708), AUC TAB vs. ultrasound (*p* = 0.829), AUC ultrasound vs. 2-[^18^F]FDG PET/CT (*p* = 0.889). adjusted for non-available and inconclusive patients.
