# Supplementary material for: Ultrasound, PET/CT or temporal artery biopsy for giant cell arteritis? A prospective diagnostic accuracy study (the GAME‐study)
Source: Acta Ophthalmol. 2026 Mar 7;104(6):e695–708. doi: 10.1111/aos.70114 (PMC13460362; doi:10.1111/aos.70114)
Supplement: Supplementary file 3 [file AOS-104-e695-s003.docx]

**Supplementary file 3.** 2 x 2 Contingency tables.

|  |  | **Temporal artery biopsy** | | **Total** |
| --- | --- | --- | --- | --- |
|  |  | **Positive** | **Negative** |  |
| **Ultrasound** | **Positive** | 25  1^a^ | 8  24 | 33  25 |
|  | **Negative** |  |  |  |
|  | **Total** | 26 | 32 | 58 |
|  |  |  |  | Persons Chi-square < 0,0001 |

|  |  | **Temporal artery biopsy** | | **Total** |
| --- | --- | --- | --- | --- |
|  |  | **Positive** | **Negative** |  |
| **2-[^18^F]FDG PET/CT** | **Positive** | 22  4^b^ | 6  26 | 28  30 |
|  | **Negative** |  |  |  |
|  | **Total** | 26 | 32 | 58 |
|  |  |  |  | Persons Chi-square < 0,0001 |

|  |  | **Ultrasound** | | **Total** |
| --- | --- | --- | --- | --- |
|  |  | **Positive** | **Negative** |  |
| **2-[^18^F]FDG PET/CT** | **Positive** | 26  7 | 2  23 | 28  30 |
|  | **Negative** |  |  |  |
|  | **Total** | 33 | 25 | 58 |
|  |  |  |  | Persons Chi-square < 0,0001 |

Bonferroni Correction original alfa level 0.05 number of comparisons 3 *p* value less than 0.01667

Upper part shows temporal artery biopsy and ultrasound. Middle part shows temporal artery biopsy and 2-[^18^F]FDG PET/CT. Lower part shows ultrasound and 2-[^18^F]FDG PET/CT. Only positive and negative tests are included all non-available and inconclusive test are removed. ^a^A patient with (GCA with A-AION, positive TAB, negative ultrasound, and 2-[^18^F]FDG PET/CT. Glucocorticoid treatment initiation before ultrasound and 2-[^18^F]FDG PET/CT were 5 and 6 days respectively). ^b^One patient with (GCA with A-AION, glucocorticoid was initiated 8 days before 2-[^18^F]FDG PET/CT), One patient with (GCA with A-AION, glucocorticoid was initiated 6 days before 2-[^18^F]FDG PET/CT), One patient with (GCA with A-AION, glucocorticoid was initiated 2 days before 2-[^18^F]FDG PET/CT), a patient with (GCA and blurred vision, glucocorticoid was initiated 4 days before 2-[^18^F]FDG PET/CT).
