# Supplementary material for: Ultrasound, PET/CT or temporal artery biopsy for giant cell arteritis? A prospective diagnostic accuracy study (the GAME‐study)
Source: Acta Ophthalmol. 2026 Mar 7;104(6):e695–708. doi: 10.1111/aos.70114 (PMC13460362; doi:10.1111/aos.70114)
Supplement: Supplementary file 4 [file AOS-104-e695-s004.docx]

**Supplementary file 4.** Table of final specific clinical diagnosis at 6 months follow-up classified as “other diagnosis” by a rheumatologist in table 1.

| **GCA** | **Non-GCA** | **Inconclusive** | **Total** |
| --- | --- | --- | --- |
| **n = 0** | **n = 22** | **n = 2** | **n = 24** |
|  | Headache |  |  |
|  | Non-available |  |  |
|  | Sinusitis |  |  |
|  | Atrial fibrillation |  |  |
|  | Occipital infarct |  |  |
|  | Ocular herpes |  |  |
|  | Non-available |  |  |
|  | Possible viral disease |  |  |
|  | Possible Tolosa-Hunt |  |  |
|  | Non-available |  |  |
|  | Arthritis |  |  |
|  | Cilioretinal occlusion |  |  |
|  | Sinusitis |  |  |
|  | Osteoarthritis |  |  |
|  | Mandibular osteomyelitis |  |  |
|  | Rheumatoid arthritis |  |  |
|  | Possible malignancy |  |  |
|  | Polymyositis |  |  |
|  | Carpal tunnel syndrome |  |  |
|  | Isolated AION |  |  |
|  |  | Visual loss |  |
|  |  | Headache |  |
|  | Pneumonia |  |  |
|  | Meningioma |  |  |

Abbreviations: n = number of patients; GCA = giant cell arteritis.
