# Supplementary material for: Ultrasound, PET/CT or temporal artery biopsy for giant cell arteritis? A prospective diagnostic accuracy study (the GAME‐study)
Source: Acta Ophthalmol. 2026 Mar 7;104(6):e695–708. doi: 10.1111/aos.70114 (PMC13460362; doi:10.1111/aos.70114)
Supplement: Supplementary file 6 [file AOS-104-e695-s006.docx]

**Supplementary file 5.** Table of specific final neuro-ophthalmological “other diagnosis/signs” in table 2.

| **Total** | **GCA**  **(n = 61)** | **Non-GCA**  **(n = 39)** | **Inconclusive**  **(n = 3)** | **Total**  **(n = 103)** |
| --- | --- | --- | --- | --- |
| **Other diagnosis, n** | **n = 29** | **n = 23** | **n = 2** | **n = 54** |
| BRAO | 1 | 1 | 0 | 2 |
| Transitory blurred vision | 9 | 4 | 0 | 13 |
| Transitory unspecified visual disturbances | 1 | 1 | 0 | 2 |
| Unspecified blurred vision | 1 | 0 | 0 | 1 |
| Blurred vision | 2 | 3 | 1 | 6 |
| Long lasting blurred vision | 2 | 1 | 0 | 3 |
| Long lasting visual scintillations | 1 | 0 | 0 | 1 |
| Long lasting unspecific visual disturbances | 0 | 1 | 0 | 1 |
| Intermittent scintillations | 1 | 0 | 0 | 1 |
| Transitory blurred vision and diplopia | 1 | 0 | 0 | 1 |
| Transitory blurred vision and scintillations | 1 | 0 | 0 | 1 |
| Transitory diplopia and scintillations | 1 | 0 | 0 | 1 |
| Transitory diplopia | 2 | 1 | 0 | 3 |
| Double vision | 1 | 1 | 0 | 2 |
| A-CRAO | 1 | 0 | 0 | 1 |
| Homonym hemianopsia | 0 | 1 | 0 | 1 |
| Homonymous quadrantanopia | 0 | 1 | 0 | 1 |
| Ocular pain | 0 | 1 | 0 | 1 |
| Meningioma compressing optic nerve causing blurred vision | 0 | 1 | 0 | 1 |
| Vision loss | 1 | 0 | 1 | 2 |
| Transient visual loss | 3 | 5 | 0 | 8 |
| Glaucomatous optic neuropathy | 0 | 1 | 0 | 1 |

Abbreviations: n = number of patients; GCA = giant cell arteritis.
