# Supplementary material for: Ultrasound, PET/CT or temporal artery biopsy for giant cell arteritis? A prospective diagnostic accuracy study (the GAME‐study)
Source: Acta Ophthalmol. 2026 Mar 7;104(6):e695–708. doi: 10.1111/aos.70114 (PMC13460362; doi:10.1111/aos.70114)
Supplement: Supplementary file 7 [file AOS-104-e695-s007.docx]

**Supplementary file 1.** Definition of GCA subtypes.

| **GCA subtype** | **Definition** |
| --- | --- |
| Large vessel | Vertebral arteries (PET and UL not routine)  Carotid arteries (PET and UL)  Subclavian arteries (PET and UL)  Axillary arteries (PET and UL)  Brachiocephalic trunk (PET)  Thoracic aorta (PET)  Internal mammary arteries (PET)  Abdominal aorta (PET)  Iliac arteries (PET)  Femoral arteries (PET) |
| Cranial | Temporal arteries (PET and UL)  Facial arteries (PET and UL)  Maxillary arteries (PET and UL)  Lingual arteries (PET and UL not routine)  Occipital arteries (PET and UL)  Ophthalmic arteries (OCT) |
| Large and cranial | Combination of the two |
| **Polymyalgia rheumatica (PMR)** | No vessel inflammation, signs of PMR |
| **Other diagnosis** | No vessel inflammation |
| **Non-available** | No vessel inflammation, non-available |

PET = Arteries where vasculitis can be visualized on 2-[^18^F]FDG PET/CT**.** UL = Arteries where vasculitis can be visualized on ultrasound. OCT = Arteries where vasculitis can be visualized on optical coherence tomography. (Adopted from van der Geest KSM, Sandovici M, Bley TA, Stone JR, Slart RHJA, Brouwer E. Large vessel giant cell arteritis. *Lancet Rheumatol*. 2024;6(6):e397-e408. doi:10.1016/S2665-9913(23)00300-4).
