# Supplementary material for: Ultrasound, PET/CT or temporal artery biopsy for giant cell arteritis? A prospective diagnostic accuracy study (the GAME‐study)
Source: Acta Ophthalmol. 2026 Mar 7;104(6):e695–708. doi: 10.1111/aos.70114 (PMC13460362; doi:10.1111/aos.70114)
Supplement: Supplementary file 8 [file AOS-104-e695-s008.docx]

**Supplementary file 2.** Baseline biomarkers.

|  | **GCA**  **(n = 39)** | **Non-GCA**  **(n = 28)** | ***p* value** | **Inconclusive**  **(n = 0)** | **Total**  **(n = 67)** |
| --- | --- | --- | --- | --- | --- |
| C-reactive protein, n  Median (IQR), mg/L  Low/Normal < 10 mg/L, n  Elevated ≥ 10 mg/L, n | 39  50.0 (19.0-90.0)  4  35 | 28  5.5 (1.4-20.3)  17  11 | <0.001^c^ | 0  -  0  0 | 67  24.0 (6.7-68.0)  21  46 |
| Erythrocyte sedimentation rate, n  Median (IQR), mm  Low/Normal ≤ 15/20 mm, n  male/female  Elevated > 15/20 mm, n  male/female | 39  53.0 (37.0-85.5)  34  5 | 28  15.0 (8.8-27.0)  10  18 | <0.001^c^ | 0  -  0  0 | 67  38.0 (14.0-69.0)  44  23 |
| Platelet count, n  Median (IQR), 10^9^/L  Low/Normal < 400x10^9^/L, n  Elevated ≥ 400x10^9^/L, n | 39  364.0 (306.5-428.0)  24  15 | 28  267.5 (213.5-294.0)  28  0 | <0.001^c^ | 0  -  0  0 | 67  311.0 (262.0-385.5)  52  15 |

Abbreviations: GCA = giant cell arteritis; IQR = Interquartile range; number of patients = n. Adjusted for non-available samples. ^c^ Mann-Whitney U test. *p* < 0.05 is statistically significant.
